# Hepatocellular Carcinoma Transplant Criteria Show Poor Negative Predictive Value: A Systematic Review and Meta-Analysis

**DOI:** 10.3390/jpm15100449

**Published:** 2025-09-24

**Authors:** Laura P. Frazão, Margarida C. Quaresma, José B. Pereira-Leal, Christophe Duvoux, Joana Cardoso

**Affiliations:** 1 Ophiomics—Precision Medicine, 1600-514 Lisboa, Portugal; laurapmffrazao@gmail.com (L.P.F.); mquaresma@ophiomics.com (M.C.Q.); jleal@ophiomics.com (J.B.P.-L.); 2 Service d’Hépatologie, Assistance Publique-Hôpitaux de Paris (AP-HP), Hôpitaux Universitaires Henri Mondor, Université Paris Est Créteil, 94000 Créteil, France; christophe.duvoux@aphp.fr

**Keywords:** HCC, liver transplantation, criteria, meta-analysis, measures of diagnostic performance, negative predictive value

## Abstract

**Background/Objectives:** Multiple criteria are used worldwide to select hepatocellular carcinoma (HCC) patients with a low risk of recurrence for liver transplantation (LT). However, it remains unclear which criteria are best for the LT-involved stakeholders, particularly in accurately identifying patients at high risk of recurrence. This work aimed to identify the most accurate criteria for selecting HCC patients for LT. **Methods:** In June 2023, a systematic literature search was conducted in PubMed and CENTRAL to identify studies including LT selection criteria of HCC patients. Data was extracted from recurrence-free survival curves using a validated algorithm and subsequently used to calculate measures of diagnostic performance routinely used in clinical trials. The Preferred Reporting Items for Systematic Reviews and Meta-Analyses (PRISMA) reporting guidelines were applied. **Results:** Of 815 records screened, only 17 met our study inclusion parameters, embodying 14 LT selection criteria. All LT criteria achieved an adjusted positive predictive value (aPPV) over 80%, indicating the correct selection of at least 80% of low-risk HCC patients. However, the adjusted negative predictive value (aNPV) was below 50% in most cases, indicating that these criteria cannot correctly identify patients with a true high risk of recurrence. This raises major ethical concerns regarding the models’ ability to exclude patients from LT. Since a perfect model is nonexistent, we created a ranking to account for the distinct concerns of all stakeholders in LT eligibility in the context of HCC. **Conclusions:** These results highlight the urgent need for refined or newly developed criteria with improved specificity and NPV to select more patients amenable to LT who are currently excluded.

## 1. Introduction

According to GLOBOCAN 2022 data, liver cancer was the sixth most diagnosed cancer, with approximately 865,000 new cases, and the third leading cause of cancer-related deaths worldwide, with nearly 758,000 deaths, reflecting a particularly poor prognosis with a mortality-to-incidence ratio of 0.88 [1]. Hepatocellular carcinoma (HCC) accounts for 75–85% of primary liver cancers and thus represents a major global health burden. Its incidence varies geographically: Eastern Asia and sub-Saharan Africa show the highest rates due to hepatitis B virus (HBV) prevalence, while Western countries face a rising incidence linked to metabolic dysfunction-associated steatotic liver disease (MASLD) and hepatitis C virus (HCV) [2].

Liver transplantation (LT) is considered the most effective curative treatment for HCC [3,4] because it addresses both the tumor and the underlying liver disease. However, LT effectiveness is constrained by critical limitations, namely organ scarcity (only 10–15% of eligible patients receive a transplant [5]), high dropout rates during the waiting periods (15–30% at one year) [6], and post-transplant HCC recurrence in 10–20% of cases despite careful selection [7]. Living donor LT (LDLT) has emerged as a partial solution to organ shortage, but due to distinct LT eligibility criteria it is associated with higher recurrence rates and donor mortality risks, making deceased donor LT (DDLT) still the preferred option globally [8].

Over the past two decades, over 20 criteria have been developed to identify HCC patients who are less likely to experience HCC recurrence and who would derive maximum benefit from LT [9,10,11,12,13,14,15,16,17,18,19,20,21,22,23,24,25,26,27,28,29,30], thereby ensuring fair and optimized organ allocation.

The Milan criteria, introduced by Mazzaferro et al. in 1996, established the foundational framework and remain the benchmark [17]. The expanded models such as the University of California San Francisco (UCSF) criteria [20], the alpha-fetoprotein score (AFP-score) [31], and Metroticket 2.0 (MT2.0) model [32] primarily rely on tumor morphology [9,17,18,20], but the newer iterations such as the AFP-score and MT2.0 also incorporate biological surrogate markers such as AFP serum levels [9,18]. These criteria successfully identify patients with acceptable post-transplant outcomes, achieving 5-year recurrence rates as low as 15% and 5-year survival rates up to 70% [26,33,34,35,36,37,38,39]. However, significant challenges persist in their implementation and validation; thus, despite the advances, no consensus exists on the optimal selection method. This absence of consensus stems from multiple factors. First, limited prospective validations in independent, multi-center cohorts reduce generalizability [40]. Second, direct comparisons between criteria within identical patient populations remain scarce [41]. Third, studies frequently include confounding variables such as combining outcomes from living donor and deceased donor liver transplantation, which have different selection thresholds and outcomes [41]. Fourth, reliance on composite endpoints such as overall survival (OS) and recurrence-free survival (RFS) introduces cohort-dependent biases that may obscure true discriminatory capacity [42]. Finally, current evaluations inadequately address the diverse priorities of key stakeholders in HCC-related LT decisions: patients (maximizing access and survival), physicians (optimizing outcomes), payers (cost-effectiveness), and organ-allocation organizations (OAOs) (equity and utility) [43].

Additionally, while existing criteria demonstrate high positive predictive value (PPV) for identifying good prognosis patients, their negative predictive value (NPV), the ability to correctly identify patients who would relapse, remains poorly defined. This limitation raises ethical concerns: the extent to which patients excluded from transplantation by current criteria would have remained recurrence-free had they received a transplant remains unknown, potentially denying life-saving treatment to salvageable patients. Therefore, an objective comparison of existing selection criteria using standardized measures of diagnostic performance (sensitivity, specificity, PPV, NPV, overall accuracy and others [44]) is essential, as recommended by the STARD (Standards for Reporting Diagnostic Accuracy) guidelines [45]. Such metrics enable objective assessment aligned with stakeholder priorities, ensuring that the accuracy ratings are directly associated with meaningful results: sensitivity reflects inclusiveness (patient perspective), specificity indicates appropriate organ utilization (allocation perspective), PPV predicts successful outcomes (physician perspective), and NPV highlights missed opportunities (ethical perspective).

This systematic review and meta-analysis aims to: (1) comprehensively evaluate and compare the diagnostic performance of established HCC selection criteria for LT using standardized metrics; (2) characterize the relevance of each measure of diagnostic performance for the different stakeholders; and (3) provide a methodological framework and performance baseline for future criteria development and validation studies.

## 2. Methods

Study Registration and Reporting Standards: This study was retrospectively registered at the Open Science Framework (OSF) under the identifier https://doi.org/10.17605/OSF.IO/2VKRW (accessed on 1 July 2025). We adhered to the Preferred Reporting Items for Systematic Reviews and Meta-Analyses (PRISMA) guidelines throughout the study design, execution, and reporting.

Literature Search Strategy: We conducted a systematic search in PubMed and CENTRAL (Cochrane Central Register of Controlled Trials) up to June 2023. The search strings used were (“Liver” OR “Hepatic”) AND (“Transplant” OR “Transplantation”) AND (“HCC” OR “Hepatocellular Carcinoma”) AND “Selection Criteria” NOT “living donor” NOT “Downstaging” NOT “Resection”. We included studies that defined LT selection criteria for patients with HCC. Additional studies were identified through citation tracking of relevant reviews and meta-analyses.

Terminology and Definitions: Recurrence-Free Survival (RFS) was defined as the time from LT to the first documented recurrence. Patients without documented recurrence at the last follow-up or who died without recurrence were censored. When terminologies/definitions were not explicitly stated, we assumed consistency with our definitions if separate plots for overall survival (OS) and RFS were presented (see Table S1).

Eligibility Criteria: Studies were selected using the PICOTS (Population, Intervention, Comparator, Outcomes, Timing, Setting) framework, as outlined in Table S2. Briefly, we included only studies with adult cohorts that underwent deceased-donor LT (less than 20% of living donors), with fewer than 50% downstaged patients before LT, a minimum follow-up of 3 years, availability of RFS or equivalent curves with patient-at-risk data and LT selection criteria had to be solely based on pre-transplant variables. Studies using LT explant pathology to define criteria were excluded.

Data Extraction and Reconstruction of Individual Patient Data (IPD): Data was extracted from recurrence/disease/tumor-free survival curves as reported elsewhere [46]. Briefly, survival data were first extracted from the published Kaplan–Meier curves of each included study using WebPlotDigitizer software (https://apps.automeris.io/wpd/ (accessed on 1 July 2023)), a computer vision-assisted software that helps extract numerical data from images. The extracted numerical data (coordinates from the curves and the number of patients at risk at specific time points) were then imported into the R programming environment. This data was used as the input for the algorithm by Guyot et al. [46], which reconstructs IPD from a published survival curve. All algorithmic analysis was performed in the R environment using the already implemented algorithm, available as an R script, from the Guyot et al. [46] original publication. The algorithm output provided individual patient data including follow-up time and recurrence status. In particular cases, when plots were presented as cumulative recurrence rate/incidence/risk, values were transformed by subtracting each coordinate from 1 before applying the algorithm. When studies included both training and validation cohorts, only the validation cohort data were used to avoid overfitting bias.

Analytical Strategy and Measures of Diagnostic Performance: Measures of Diagnostic Performance were calculated as outlined in Figure S1. We calculated sensitivity, specificity, positive predictive value (PPV), negative predictive value (NPV), and overall accuracy at 3 and 5 years post-LT. To ensure comparability across cohorts with varying recurrence rates, PPV, NPV, and accuracy were adjusted to a fixed event prevalence of 0.87, the average of no-recurrence prevalence reported in the literature [26,33,34,35,36,37,38,39]. Adjusted metrics can be modified or recalibrated to account for other factors besides the test’s inherent characteristics (sensitivity and specificity), such as the disease’s prevalence in a specific population. The adjusted PPV, NPV, and accuracy more accurately reflect the real-world predictive values for a given group or situation. This adjustment allows for a direct comparison of measures of diagnostic performance across different cohorts and time points. For criteria with multiple datasets, measures of diagnostic performance were calculated independently per dataset, and their means and respective standard deviations were reported.

Risk of Bias Analysis and Quality Assessment: Each included study was assessed using two tools: the Risk of Bias in Cohort Studies (RBCS) tool [47] and the Quality Assessment of Prognostic Accuracy Studies Tool (QUAPAS) tool [48]. Adaptation of these tools for this study are detailed in Table S3. Assessments were performed independently by two reviewers, with discrepancies resolved by consensus.

Software usage: WebPlotDigitizer software (https://apps.automeris.io/wpd/ (accessed on 1 July 2023), version 4.6.), R programming environment version R 4.3.1 (July 2023). All data analysis was performed in the R environment using standard functions available from distinct R packages such as MASS: (v7.3-60), splines: (v4.3.1), survival (v3.5-7). Plots were generated using standard Microsoft Excel plotting functions.

## 3. Results

### 3.1. Literature Search Results

**Figure 1:**
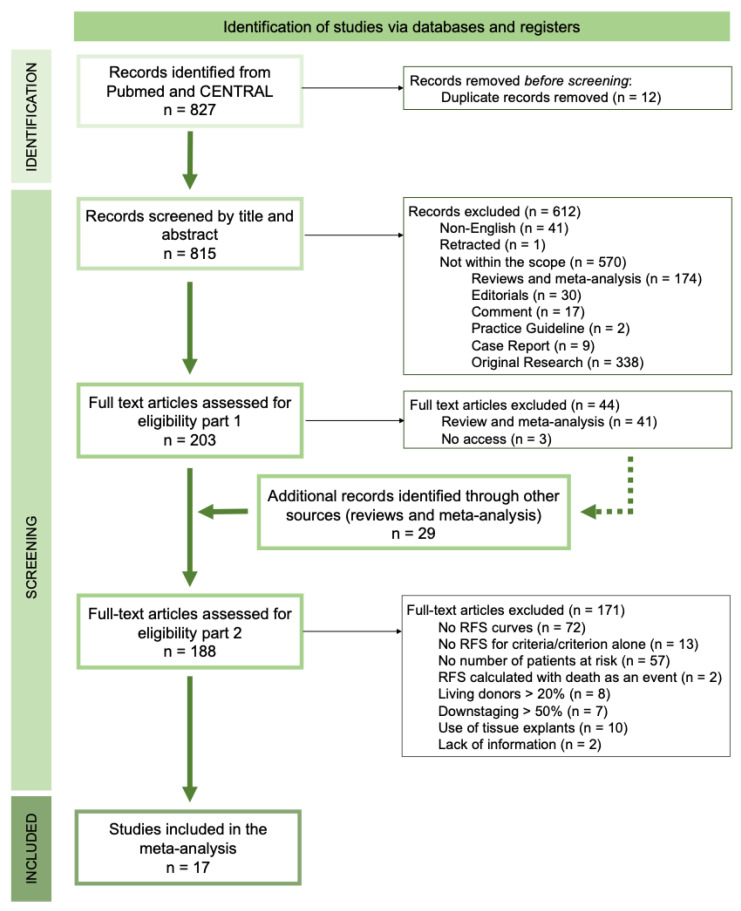
The PRISMA flow diagram illustrates the systematic process of study selection for inclusion in the analysis. The process is structured into three key phases: 1. Identification of potentially relevant records; 2. Screening of these records for eligibility according to the defined inclusion and exclusion criteria by applying detailed multiple exclusion filters; 3. The included records were finally compiled and analyzed to be included in the present study. Ultimately, 17 studies met all the criteria and were included in the final analysis. Detailed information regarding each record assessed is available in Supplementary Material (Table S1). RFS—Recurrence-free survival.

A total of 827 records were screened by title and abstract. After removing duplicates (n = 12), non-English language studies (n = 41), one retracted study, and studies outside the intended scope (n = 570), 203 full-text articles were assessed. Among these, inaccessible studies (n = 3) and reviews and meta-analyses (n = 41) were excluded, although their citations were screened for additional eligible records (n = 29). In total, 188 full-text articles were screened for eligibility (Figure 1).

Among the eligible records, 67% (n = 125) reported OS curves, while 55% (n = 103) reported RFS Kaplan-Meyer (KM) curves for a single criterion. Only 4% (n = 8) of the studies compared criteria performance using measures of diagnostic performance, and just 1% (n = 2) reported the follow-up time. Of the 103 studies with RFS curves for a single criterion, only 45% (n = 46) met our eligibility standards by providing the number of at-risk patients (Table S1). From these 46 studies, several were excluded due to methodological limitations: 2 (4.4%) calculated RFS with death as an event, 8 (17.4%) included over 20% of living donors, 7 (15.2%) had a majority of downstaged patients, 10 (21.7%) relied on variables not available at the time of LT eligibility assessment (i.e., data from tumor explant), and 2 (4.4%) did not clearly report inclusion/exclusion criteria. Ultimately, 17 studies met all inclusion criteria and were included in the meta-analysis, representing 2% of the initially screened records, 8% of the full-texts assessed, and 37% of those reporting RFS curves with at-risk counts (Figure 1).

### 3.2. Risk of Bias Assessment

Quality assessment of the 17 included studies was performed using both RBCS and QUAPAS tools (Table S3). As summarized in Table S4 and Figure S2, most studies showed a low risk of bias. However, for domains 7 and 12, 53% and 20% of the studies, respectively, had a moderate or high risk of bias. This indicates that most studies (73%) did not follow patients for a sufficient amount of time. In 50% of the studies, censored cases had follow-up shorter than 3 years and accounted for 30–60% of the cohort, while in 20% of studies they represented more than 60% of the cohort. Additionally, concerning domain 8, 60% of the studies did not report information on pre-LT and post-LT therapies across groups (within and outside criteria), limiting the assessment of potential bias.

### 3.3. Cohorts

This meta-analysis reviewed 17 studies to identify criteria for selecting patients who would benefit from LT. Of the 14 criteria analyzed, data for 11 (all except Milan, AFP score and MT2.0) came from a single study. Table 1 summarizes the details of each criterion, and Table S5 provides detailed descriptions of the included studies. Notably, well-known criteria like HALT-HCC (Hazard Associated with Liver Transplantation for Hepatocellular Carcinoma) [49], pre-MORAL (Model of Recurrence After Liver Transplant) [11], and NYCA (New York/California Score) [12,13] could not be included because they did not meet the inclusion criteria.

The LT selection criteria were at different stages of development. Some had been tested in independent cohorts (Milan, UCSF, Shanghai, AFP Score, Up7, MT2.0, Hangzhou, wALL) [21,25,26,29,50,51,52,53,54,55], while others were in the validation phase (ArgScore and Warsaw criteria [24,25]) or training phase (PLR, AFPdelta and GGT criteria [22,23,27]) (Table 1).

Overall, data from 8032 patients was included. Cohort sizes varied, and patient numbers decreased over time due to censored events (patients who died without recurrence or were lost to follow-up). The recurrence rates varied greatly between cohorts (10–57%) and increased with longer follow-up (Figure S3).

### 3.4. Measures of Diagnostic Performance

Measures of diagnostic performance were calculated from RFS curves at 3- and 5-year endpoints, excluding censored cases. Measures were also calculated in total, i.e., independently of follow-up time and including censored cases. To better reflect real-world scenarios, prevalence-dependent measures were normalized to a fixed no-recurrence prevalence reported in the literature (0.87) [26,33,34,35,36,37,38,39]; pre-normalization values are displayed in Figure S4.

**Figure 2:**
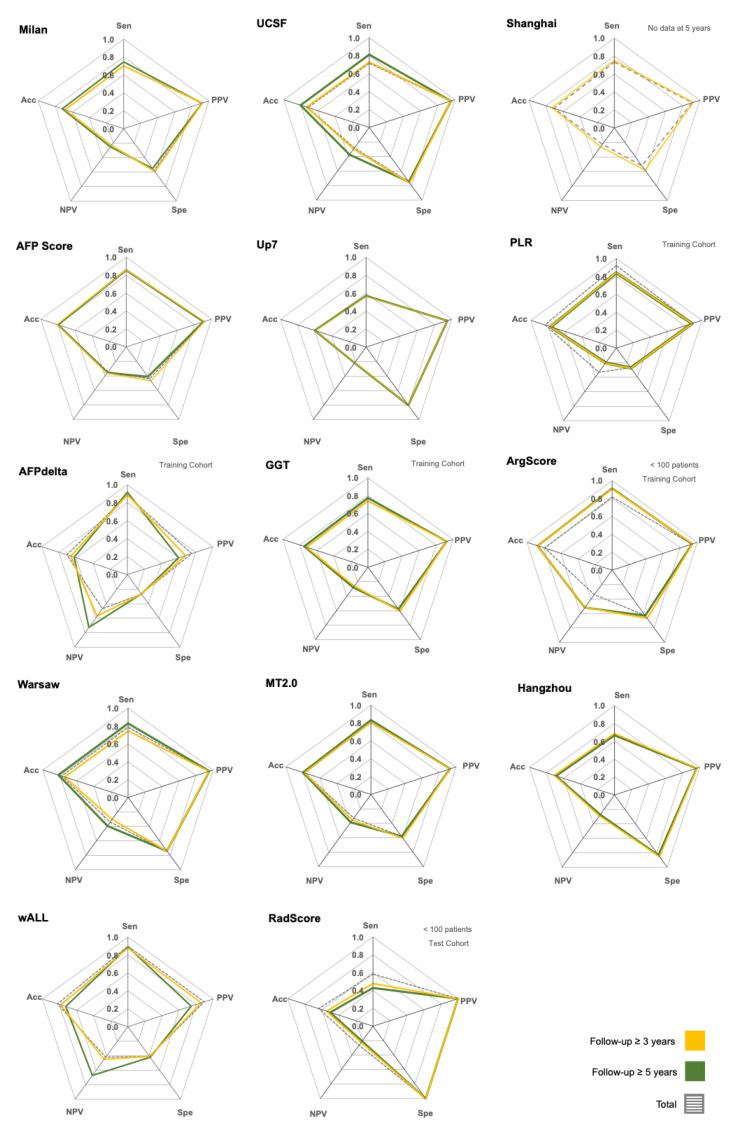
Radar charts comparing diagnostic performance measures of clinical criteria for predicting recurrence-free survival. Each chart represents a distinct criterion, evaluated across five key measures of diagnostic performance: Sensitivity (Sen) adjusted PPV (PPV), specificity (Spe), adjusted NPV (NPV), and adjusted accuracy (Acc). Adjusted values were calculated using a mean no-recurrence prevalence of 0.87, as reported in the literature. The criteria assessed include Milan, UCSF, Shanghai, AFP Score, Up7, PLR, AFPdelta, GGT, ArgScore, Warsaw, MT2.0, Hangzhou, wALL, and RedScore. Performance is visualized for two follow-up durations: up to 3 years (yellow line polygons) and up to 5 years (green line polygons). The shape and area of each polygon reflect the relative strength of each criterion across the five metrics, allowing for an intuitive visual comparison. Larger and more balanced polygons indicate superior and consistent predictive performance across both short- and long-term follow-up periods. UCSF—University of California, San Francisco; ArgScore—argentinian score; MT2.0—metroticket 2.0; GGT—gamma-glutamyltranspeptidade; RadScore—radiological score; Up7—up to seven; wALL—within all; AFP—alpha-fetoprotein; AFPdelta—AFP delta slope; PLR—platelet to lymphocyte ratio.

Within each criterion, no clear differences were observed in the measures of diagnostic performance across time points (Figure 2). Most relapses occurred within the first three years after LT (Table S5). Across all criteria, the NPV consistently tended to be lower than the other measures (Figure 2 and Figure S4).

### 3.5. The Best Criteria to Include Patients with a Low Risk of Recurrence

Identifying patients with good prognosis, i.e., those unlikely to relapse (true positives), while minimizing their exclusion (false negatives) is crucial. This requires high sensitivity/recall (capturing all patients who will not relapse) and a high PPV/precision (ensuring that all patients within the criteria truly will not relapse).

**Figure 3:**
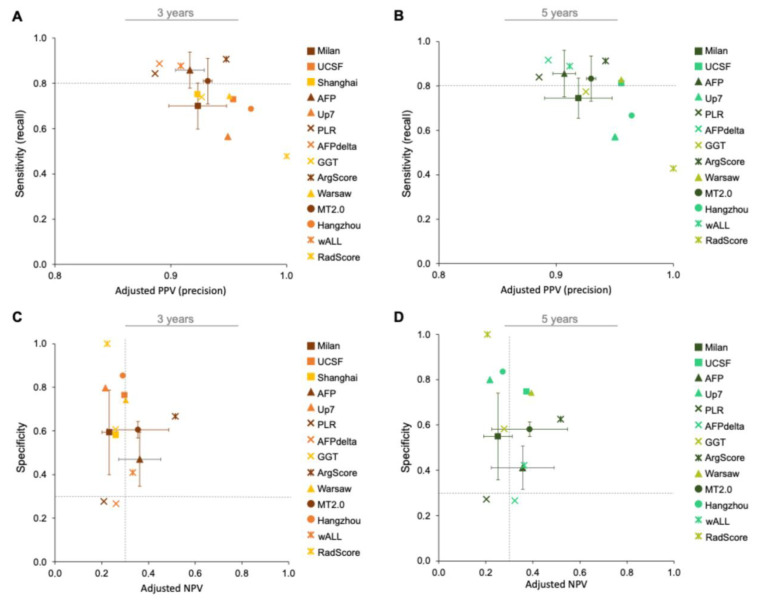
Comparative analysis of clinical criteria for including and excluding patients based on prognosis following liver transplantation (LT). The four scatter plots evaluate the predictive performance of each criterion over two follow-up periods: 3 years and 5 years. The best criteria to include patients with good prognosis after LT are characterized by high sensitivity and adjusted PPV at 3 years (**A**) and 5 years (**B**) of follow-up. In contrast, the best criteria to exclude patients with bad prognosis after LT are characterized by high specificity and adjusted NPV at 3 years (**C**) and 5 years (**D**) of follow-up. All measures of diagnostic performance were calculated considering the mean of no-recurrence prevalence described in the literature (0.87). Markers labeled with (X) denote criteria derived from cohorts with fewer than 100 patients or from training/validation datasets. indicating limited generalization. UCSF—University of California, San Francisco; ArgScore—argentinian score; MT2.0—metroticket 2.0; GGT—gamma-glutamyltranspeptidade; RadScore—radiological score; Up7—up to seven; wALL—within all; AFP—alpha-fetoprotein; AFPdelta—AFP delta slope; PLR—platelet to lymphocyte ratio.

When giving equal importance to sensitivity and adjusted PPV, only the ArgScore criterion achieved both measures above 0.90 at 3- and 5-year endpoints (Figure 3A,B), making it the best option. The wALL, AFPdelta, AFP score, PLR, and MT2.0 criteria followed, each presenting both measures above 0.80 at both endpoints.

### 3.6. The Best Criteria to Exclude Patients with a High Risk of Recurrence

To correctly identify patients with poor prognosis, it is necessary to maximize the exclusion of those at risk of relapse (true negatives) while minimizing their inclusion (false positives). Criteria with high specificity (accurately identifying patients who will relapse outside criteria) and high NPV (ensuring that most patients outside the criteria will indeed relapse) are best for this purpose.

The ArgScore was the top-performing criterion for excluding high-risk patients, with both specificity and adjusted NPV above 0.50 (Figure 3C,D). It was followed by UCSF, AFP score, wALL, Warsaw, and MT2.0 criteria, all showing specificity and adjusted NPV of at least 0.30, at both 3- (Figure 3C) and 5-year endpoints (Figure 3D).

### 3.7. Best Criteria Combining Inclusion and Exclusion of Patients for LT

To define the best criteria for selecting patients for LT, both the inclusion of low-risk patients and the exclusion of high-risk patients should be optimized, a concept captured by adjusted accuracy (aAcc). The ArgScore has the highest aAcc (0.88), followed by wALL, AFPdelta, and AFP score, each with aAcc ≥ 0.80 at both endpoints (Figure 2).

### 3.8. The Best Criteria for Meeting the Different Stakeholders’ Needs in LT

**Figure 4:**
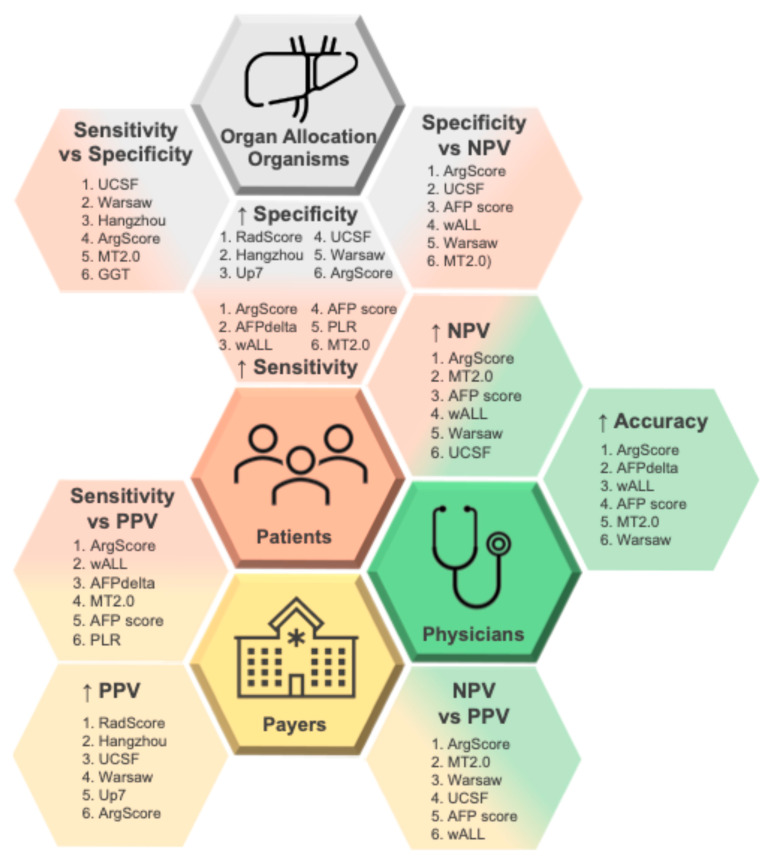
Measures of diagnostic performance contextualized within the clinical decision-making landscape of liver transplantation (LT) for hepatocellular carcinoma (HCC). The diagram maps the relevance of various predictive metrics—Sensitivity, Specificity, Positive Predictive Value (PPV), Negative Predictive Value (NPV), and Accuracy—to four key stakeholder groups: patients, physicians, payers, and Organ Allocation Organisms. The figure also highlights the top six performing criteria at both 3-year and 5-year follow-up after LT intervals for each accuracy parameter. NPV—negative predictive value; PPV—positive predictive value; UCSF—University of California, San Francisco; ArgScore—argentinian score; MT2.0—metroticket 2.0; GGT—gamma-glutamyltranspeptidade; RadScore—radiological score; Up7—up to seven; wALL—within all; AFP—alpha-fetoprotein; AFPdelta—AFP delta slope; PLR—platelet to lymphocyte ratio; up arrow (↑)—increased.

Lastly, we examined the relevance of each accuracy measure for the main stakeholders in LT for HCC: patients, physicians, payers, and organ allocation organizations (OAOs). Figure 4 shows the associations between different measures of diagnostic performance and the stakeholders’ priorities, along with the top six criteria (top6) for each category.

Physicians emphasize high overall accuracy to guide confident eligibility decisions. Patients prioritize high sensitivity to ensure that most low-risk candidates are not excluded from transplantation. Payers, such as hospitals and insurance systems, value high PPV to ensure that most patients receiving LT truly benefit from the procedure. OAOs, constrained by limited organ availability, prioritize high specificity to prevent organ allocation to high-risk patients.

Where patient and OAO priorities intersect, the most suitable criteria are those balancing specificity and sensitivity (Figure S5A,B). To address the shared concerns of patients and physicians about denying LT to high-risk patients, criteria balancing specificity and NPV are the most relevant (Figure S5C,D).

Across stakeholders, ArgScore and Warsaw criteria consistently appear among the top-ranked criteria. Considering all stakeholders together, the top6 criteria that best meet the combined priorities are ArgScore, MT2.0, Warsaw, UCSF, wALL, and AFP score (Figure 4).

## 4. Discussion

This meta-analysis is the first to systematically apply measures of diagnostic performance to compare the prognostic value of various criteria for selecting HCC patients for LT with deceased donors. The results indicate that no criterion performs perfectly; rather, their feasibility depends on the perspectives of the stakeholders involved and on the clinical context. Current international guidelines emphasize sensitivity and/or PPV to maximize inclusion of patients, reflecting ethical concerns about denying access to LT. This preference was evident in our results, where NPV consistently showed the lowest performance. The low NPV across all analyzed criteria indicates that a substantial proportion of HCC patients who could benefit from LT are still wrongly excluded, which is unacceptable from both the patient’s and the physician’s perspectives. These gaps highlight the urgent need for new predictive models with improved specificity and NPV. Ideally, such tools should balance the concerns of all stakeholders, rather than privileging only one dimension of accuracy.

Our strict inclusion standards enabled a direct comparison of performance across many currently implemented criteria. When all stakeholders’ priorities were considered together, the six best performing criteria were ArgScore, MT2.0, Warsaw, UCSF, wALL, and AFP score. Notably, the widely adopted Milan criterion was not included in the top6 [32]. This suggests that the benchmark role of Milan criteria should be reconsidered in favor of more recent and validated alternatives such as UCSF, wALL, MT2.0, or AFP score. While ArgScore and Warsaw criteria also presented good performance, their results should be interpreted with caution since they lack validation in independent datasets (only one validation or one training).

Each score offers advantages and limitations depending on the clinical context. In settings of severe organ shortage, higher specificity is most valuable, as it minimizes allocation to high-risk patients. By contrast, in contexts where organ availability is expanding, sensitivity becomes more relevant to avoid unjustly excluding candidates who would benefit. High PPV is particularly advantageous in payer-driven systems where demonstrating benefit for transplanted patients is essential. These differences explain why ArgScore and Warsaw, which balance specificity and sensitivity, consistently rank highly, while AFP score and wALL gain value in settings that prioritize broader inclusion.

We focused on RFS over OS as the outcome event because it is directly linked to poor patient survival [37,38,39], places a higher cost on healthcare systems, and avoids confounding by non-cancer-related mortality. However, the availability and quality of RFS reporting were limited: only 61.7% of the eligible studies included RFS KM curves, and less than half of these (37.9%) included the number of at-risk patients in at least three different time points, being sufficient for data extraction. Of these studies, only 36.4% were included, as the others had confounding variables such as a living donor rate above 20%, downstaging cases above 50%, or relied on data from tissue explants. Consequently, well-known criteria such as HALT-HCC [49], pre-MORAL [11], and NYCA [12,13] were excluded from this meta-analysis. This underlines the need for more consistent and transparent reporting of RFS outcomes, with clear discrimination of the number of patients at risk at different time points and/or explicit event definitions, to enable comprehensive comparisons.

The included studies were also heterogeneous in sample size (64–969 patients) and recurrence rates (10–57%), reflecting differences in populations, clinical practices and follow-up protocols. Although we adjusted prevalence-dependent measures to a standardized no-recurrence prevalence (0.87 [26,33,34,35,36,37,38,39]), this variability inevitably influences the reported performance of each criterion. The results of our meta-analysis should therefore be interpreted with caution.

Despite these limitations, several key insights emerge. First, measures of diagnostic performance provide an objective framework for comparing criteria and aligning them with stakeholder priorities. Second, specificity and NPV represent clinical gaps that must be addressed to improve patient selection. This is particularly relevant in regions where increasing organ availability (e.g., in Spain and Italy, due to the reduction in LT indications such as hepatitis C [56,57,58]), is likely to encourage broader application of expanded criteria. Our findings suggest that such expansion should be accompanied by the refinement of current prognostic tools.

Future improvements will likely come from integrating morphologic assessment with tumor HCC biology, which has already been shown to improve outcomes after LT [59]. Several studies exploring explant-based biology, have highlighted the potential prognostic value of distinct biological features such as allelic imbalance [60], gene [10] and micro-RNA [14,61,62] expression profiles, and evolutionary distance [15]. These molecular features may be further supported by integration with AI approaches (e.g., machine learning) allowing for the analysis of large amounts of data to create prognostic algorithms optimized for specific measures of diagnostic performance [63,64]. An example is the HepatoPredict tool, which outperforms Milan and several other criteria by combining morphologic and molecular tumoral features through a machine-learning algorithm [10]. Moving forward, there is an urgent need both to independently validate promising existing criteria and/or to develop new ones focusing on the correct exclusion of high-risk patients, complementing the existing inclusion-oriented approaches.

## 5. Conclusions

As expanded LT criteria are increasingly adopted, a broader pool of patients will become eligible for LT. This shift underscores the urgent need to refine existing LT criteria or to develop new ones aiming at treatment equity (e.g., by incorporating prognostic elements of tumor biology).

## Figures and Tables

**Table 1:** Data used in the meta-analysis structured by selection criteria.

Criteria	Criteria Description	N. of Studies	Type of Dataset	Number of Patients *			Ref.
				Total	Follow-Up 3 Years	Follow-Up 5 Years	
Milan	1 tumor with diameter ≤ 5 cm or ≤3 tumors each with diameter ≤ 3 cm, and no macrovascular invasion.	10 **	Test	6399	4077	2083	[21,25,26,30,50,51,52,53,54,55]
UCSF	1 tumor with diameter ≤ 6.5 cm, or ≤3 nodules with the largest lesion with diameter ≤ 4.5 cm and total tumor diameter ≤ 8 cm.	1	Test	196	137	107	[26]
Shanghai	1 tumor with diameter ≤ 9 cm or ≤3 tumors with the largest diameter ≤ 5 cm and total tumor diameter ≤ 9 cm. No macrovascular invasion, lymph node invasion, and extrahepatic metastasis.	1	Test	969	956	No data	[21]
AFP Score	Largest tumor diameter: ≤3 cm [0 points]; 3–6 cm [1 point]; >6 cm [4 points] + Number of tumors: 1–3 [0 points]; ≥4 [2 points] + AFP level: ≤100 ng/mL [0 points]; 100–1000 ng/mL [2 points]; >1000 ng/mL [3 points]. Score ≤ 2.	6	Test *^a^	4633	2644	1537	[9,30,51,52,53,55]
Up7	Sum of the tumor number and diameter of the largest tumor ≤ 7. No microvascular invasion.	1	Test	210	43	36	[29]
PLR	Platelet to lymphocyte ratio (PLR) < 125	1	Train	343	243	243	[22]
AFPdelta	Calculation of the delta slope value using 3 different AFP measures performed at different times: (1) AFP and time of diagnosis; (2) AFP and time immediately before the last LRT; (3) AFP and time immediately before LT	1	Train	106	83	66	[27]
GGT	Gamma-glutamyltranspeptidade (GGT) ≤ 128 U/L	1	Train	130	129	129	[23]
ArgScore	AFP > 100 ng/mL [Yes = 1 point, No = 0 point], tumor beyond Up-to-7 [Yes = 1 point, No = 0 point}. Score = 0 points [low risk].	1	Validation	87	47	39	[30]
Warsaw	Expansion of Milan criteria including cases outside Milan criteria but within UCSF or Up to Seven (Up7) criteria with AFP < 100 ng/mL.	1	Validation	240	113	72	[25]
MT2.0	Number of tumors and largest tumor diameter ≤ 7 cm + AFP < 200 ng/mL; Number of tumors and largest tumor diameter ≤ 5 cm + AFP < 400 ng/mL; Number of tumors and largest tumor diameter ≤ 4 cm + AFP < 1000 ng/mL.	3	Test *^a^	793	634	478	[18,26,55]
Hangzhou	Total tumor diameter ≤ 8 cm, or Total tumor diameter > 8 cm with histopathologic grade I or II and AFP ≤ 400 ng/mL.	1	Test	196	137	106	[26]
wALL	Combination of the AFP Score and MT2.0.	1	Test	2444	1486	984	[30]
RadScore	Radiomics signature based in 7 features.	1	Test	64	50	35	[26]

* Sum of all patients from different cohorts. ** For follow-up 3 years after LT. For 5 years after LT, n = 8. *a Most test cohorts, one validation cohort. UCSF—University of California, San Francisco; AFP—alpha-fetoprotein; Up7—Up to Seven; PLR—platelet to lymphocyte ratio; LRT—Locoregional therapy; LT—liver transplantation; GGT—gamma-glutamyl transpeptidase; MT2.0—Metroticket 2.0; RadScore—radiologic score; Ref.—references.

## Data Availability

The data analyzed during the current study are available from the corresponding author upon reasonable request.

## Supplementary Materials

The following supporting information can be downloaded at: https://www.mdpi.com/article/10.3390/jpm15100449/s1, Figure S1: Accuracy Measures Calculation; Figure S2: Assessment of Risk of Bias of all studies included in the meta-analysis; Figure S3: Study Population; Figure S4: Accuracy measures for each criterion without normalizations; Figure S5: The best criterion for patients’ classification; Table S1: Description of all records analyzed for the present meta-analysis; Table S2: PICOTS strategy used to select literature studies. Studies not meeting these criteria, duplicate publications, studies in languages other than English or including post-transplant variables were excluded; Table S3: Adaptation of the Cochrane tool to assess the risk of bias in Cohort Studies and the Quality Assessment of Prognostic Accuracy Studies Tool (QUAPAS) in the present meta-analysis; Table S4: Assessment of risk of bias of the studies included in the meta-analysis; Table S5: Detailed description of the studies included in the meta-analysis.

## Abbreviations

Acc	Accuracy
AFP	Alpha-fetoprotein
AFPdelta	AFL delta slope
ArgScore	Argentinian Score
GGT	Gamma-glutamyltranspeptidase
HCC	Hepatocellular Carcinoma
KM	Kaplan-Meyer
LT	Liver Transplantation
MT2.0	Metroticket 2.0
NPV	Negative Predictive Value
OAOs	Organ-allocation Organizations
OS	Overall Survival
PLT	Platelet to Lymphocyte Ratio
PRISMA	Preferred Reporting Items for Systematic Reviews and Meta-Analyses
PPV	Positive Predictive Value
QUAPAS	Quality Assessment of Prognostic Accuracy Studies Tool; RadScore, Radiological Score
RBCS	Tool to Assess the Risk of Bias in Cohort Studies
RFS	Recurrence-free Survival
Sen	Sensitivity
Spe	Specificity
UCSF	University of California San Francisco
Up7	Up to Seven
wALL	Within All
